# Banning traditional birth attendants from conducting deliveries: experiences and effects of the ban in a rural district of Kazungula in Zambia

**DOI:** 10.1186/s12884-016-1111-9

**Published:** 2016-10-21

**Authors:** Chilala Cheelo, Selestine Nzala, Joseph M. Zulu

**Affiliations:** 1 Department of Public Health, Section of Health Promotion and Education, School of Medicine, University of Zambia, Lusaka, PO Box 50110, Zambia; 2 Minstry of Health, Kazungula District, Zambia; 3 Departments of Public Health, Section of Health Policy and Management, School of Medicine, University of Zambia, Lusaka, PO Box 50110, Zambia

**Keywords:** Traditional birth attendants, Home deliveries, Health facility deliveries, Zambia

## Abstract

**Background:**

In 2010 the government of the republic of Zambia stopped training traditional birth attendants and forbade them from conducting home deliveries as they were viewed as contributing to maternal mortality. This study explored positive and negative maternal health related experiences and effects of the ban in a rural district of Kazungula.

**Methods:**

This was a phenomenological study and data were collected through focus group discussions as well as in-depth interviews with trained traditional birth attendants (tTBAs) and key informant interviews with six female traditional leaders that were selected one from each of the six zones. All 22 trained tTBAs from three clinic catchment areas were included in the study. Content analysis was used to analyse the data after coding it using NVIVO 8 software.

**Results:**

Home deliveries have continued despite the community and tTBAs being aware of the ban. The ban has had both negative and positive effects on the community. Positive effects include early detection and management of pregnancy complications, enhanced HIV/AIDS prevention and better management of post-natal conditions, reduced criticisms of tTBAs from the community in case of birth complications, and quick response at health facilities in case of an emergency. Negatives effects of the ban include increased work load on the part of health workers, high cost for lodging at health facilities and traveling to health facilities, as well as tTBAs feeling neglected, loss of respect and recognition by the community.

**Conclusion:**

Countries should design their approach to banning tTBAs differently depending on contextual factors. Further, it is important to consider adopting a step wise approach when implementing the ban as the process of banning tTBAs may trigger several negative effects.

## Background

Poor standards of maternal and newborn care during antepartum, peripartum and postpartum period significantly contribute to the annual estimated 289,000 maternal deaths globally [1–3]. Among the eight Millennium Development Goals (MDGS) adopted at the Millennium summit is the reduction of maternal mortality ratio (MMR) by three quarters between 1990 and 2015 (MDG 5). Key to this reduction is the utilization of antenatal, delivery and postnatal services. The current maternal mortality ratio in Zambia stands at 398 per 100,000 live births which is still high compared to the MDG 5 target of reducing to 165 per 100,000 by 2015 [4]. The Every Newborn Action Plan (ENAP) impact framework inserted “*Every Newborn*” into the “*Every Woman, Every Child*” concept, broadening its goals to include ending preventable deaths for women, newborns and children, and improving child development and human capital beyond the close of the MDGs [5].

One of the factors that has affected meeting of the MDG 5 is inadequate number of skilled health workers. Several low and middle income countries (LMICs) in the world, Zambia inclusive, face critical shortages of human resources for health (HRH) [6, 7]. Factors that have contributed to this gap include the limited capacity by countries to train staff, and international migration of skilled workers in search of professional development and better quality of life [8]. This shortage of health workers has increased workload for the few health workers in LMICs, a situation which has contributed towards low work performance and de-motivating staff [9]. This shortage has greatly affected delivery of services, including maternal and child health services in the affected countries [10].

In an attempt to resolve the human resources for health gaps, countries have come up with different strategies, with one of them being the use of community based health workers such as trained traditional birth attendants to deliver health services [11]. Maternal and child health services are among some of the key health services provided by the community based health workers [12, 13].

The World Health Organization (WHO) defined a traditional birth attendant (TBA) as a person who assists the mother during childbirth and who initially acquires skills by delivering babies herself or through an apprenticeship to other TBAs. Whereas a trained traditional birth attendant (tTBA) is any TBA who has received a short course of training through the modern health sector to acquire more skills [14]. The main goal of TBA training was to reduce maternal and child morbidity and mortality [15, 16].

### Human resources crisis in Zambia and TBAs

By 1985 more than 52 countries were running training programmes for TBAs and Zambia was one of them [17, 18]. Zambia has only about half the health workforce that it needs for all the categories [19]. Vacancies among the nursing staff are 55 %, clinical officers 63 % and doctors 64 % [19]. As a result of this shortage, Zambia like many other countries realized the need to adopt and train TBAs as an interim measure until every woman was attended to by skilled personnel [20]. Therefore in 1973, Zambia initiated the training program for TBAs [21, 22]. The TBAs were trained because they were a familiar part of the birthing process worldwide [23]. In some cases, even with availability of trained personnel and health facilities, women still chose the services of TBAs because they are easily accessible, acceptable, affordable and are perceived as culturally appropriate and respectful [24].

Many studies on the impact of TBA training globally had however, indicated little or no maternal mortality reduction for which they were intended [25]. Therefore, Zambia in 2010 following the global redirection stopped the training of TBAs and forbade them from conducting home deliveries [20]. With such a background, this study aimed to explore the positive and negative maternal health related experiences and effects of the ban of home deliveries conducted mostly by trained traditional birth attendants (tTBAs) in Kazungula district, Southern Province, Zambia.

## Methods

### Research design

The study adopted a phenomenological study approach which is a recognized qualitative method. A phenomenological study approach was helpful in getting in-depth insight into the effects, experiences and meanings made out of the ban by trained traditional birth attendants and traditional leaders [6]. In-depth interviews, focus group discussions and key informant interviews using semi-structured interview guides were used to obtain information from a variety of respondents which resulted in data triangulation by contrasting and validating the data for different and similar findings [6].

### Study site

The study was conducted in Mukuni chiefdom in Kazungula district of southern province. The district was purposively selected owing to relatively low numbers of skilled and institutional deliveries (40.7 %) and high numbers of home deliveries (by tTBAs and other relatives). Kazungula district has a total population of 113,666 with 22 health facilities [26]. The district has no hospital but cases were referred to Livingstone and Mwandi hospital. All 22 facilities offer antenatal and postnatal services while delivery services are not offered in some facilities [26].

### Study population

Only traditional birth attendants who were trained (tTBAs) and recognized by government and resided in Mukuni chiefdom were included. In addition, female traditional leaders who are the custodians of all matters related to women including reproductive health issues in the chiefdom were included. This provided a homogeneous sample based on shared lived experiences.

### Data collection

#### Key informant interviews

An expert sample for key informant interviews (in-depth) comprised of all female traditional leaders (banabedyango) from the six zones namely Gundu, Libala, Kayube, Makoli, Mulindi, and Manyemunyemu zones. These informants were selected based on the fact that they were members of the same group (banabedyango) and hold important views on traditional matters related to women including maternal health issues in their zones.

#### Focus group discussions

A total of three (3) focus group discussions (FGDs) were conducted with all the 22 trained traditional birth attendants. The first FGD was held at Manyemunyemu area with 8 participants. The second FGD which was held at Katapazi area had 7 participants and the final FGD was conducted in Mukuni area with 7 participants. These were audio-recorded.

#### In-depth interviews

Nine in-depth interviews were conducted with the same tTBAs that participated in the FGDs in order to have additional information which may not have been brought out in the FGDs. Maximum variation sampling was used to select participants from the trained traditional birth attendants to take part in in-depth interviews. Three (3) participants from each catchment area (total 9 participants) were selected based on the highest number of experience, the medium and the lowest in order to triangulate the sources of information and help generate in-depth unique insights and shared patterns that cut across the *duration of experience*. An interview schedule was prepared and used during the interviews containing unstructured/open ended questions. The interviews were conducted at a place that was conducive to the respondents, in their homes.

### Data analysis

Data analysis was done using qualitative content analysis, following Graneheim and Lundman [27]. The process of data analysis started with the reading of field notes and listening to recorded interviews. The interviews were then transcribed by verbatim. Having completed the transcription process, we read through the interviews many times to capture the context and meaning. Transcripts were then analysed for identification of text (meaning units) related to positive and negative experiences of the ban of traditional birth attendants. The meaning units were condensed into codes, and similar codes were then grouped together into categories and finally grouped into themes [27].

### Ethical considerations

Ethical clearance was sought from the Excellency in Research Ethics and Science (ERES) committee, reference number 2014-May-020. Written permission to conduct the study was sought from relevant authorities at the Ministry of Health and Kazungula district health office. Oral permission was also obtained from his royal highness Senior Chief Mukuni. Both oral and written consent were obtained from study participants. Participants were assured of anonymity since no names were written on the interview guides except serial numbers and key informants were interviewed individually, privately and from their homes. Permission was sought from the participants to use a tape recorder.

## Results

### Attributes of TBAs

In this study, the trained traditional birth attendants (tTBAs) had varying attributes among them that qualified them to attend to mothers. The common attributes were maturity by age and years of experience. Table 1 below provides more details on the attributes of the tTBAs.

**Table 1:** Characteristics of TBAs and female traditional leaders (FTL)

Characteristics	Number of participants	
Age (TBAs)		(FTL)
30–34	4	
35–39	5	
40–44	6	3
45–49	2	1
50–54	2	1
55–59	1	
60–64	1	1
65+	1	
Total	22	6
Years of experience		
1–5	3	2
6–10	7	2
11–15	5	1
16–20	4	
21–25	2	1
25+	1	
Total	22	6

### Perceived reasons for the ban

The trained traditional birth attendants and all the key informants were all aware of the decision by government to stop home deliveries. The two groups were not officially informed about the reasons of the ban. They thought the ban aimed at reducing HIV transmission and pregnant related complications, preventing maternal and infant deaths, as well as help reduce delays in making decisions at community level. Below the perceived reasons for the ban are explained in detail:

*“I just thought that maybe it is because of this HIV/AIDS that has gone so much into the communities. Government was trying to reduce the spread of the disease from mothers to their babies during delivery because the people that used to conduct these deliveries were not fully knowledgeable on how to prevent the same disease. Otherwise it is just thinking we were not given any reasons why the decision was made”* (Key Informant 5).

*“As traditional birth attendants we have no much knowledge on how to handle complicated cases like when the child is breech so at the clinic they know how to handle such cases. Sometimes the child maybe born with a disease like being born with a yellow skin and we don’t know what to do in such cases, all we know is how to deliver” (*Respondent 2, FGD 1).

*“The reasons I was told were many including the need for reduction of infant and maternal deaths and many other complications that are common in the community”* (30-year old tTBA).

*“We are trained but we do not think we have the skills like those of nurses and doctors and we don’t have the equipment to use except the foetoscopes”* (Respondent 1, FGD 1).

*“Even when there is a complication some people delay to make decisions to take women to the hospital due to for example dependence on community deliveries….”* (Respondent 6, FGD 1).

### Positive effects of the ban

The positives effects of the ban included early detection of complications and faster transportation of women to health facilities for further management, enhanced hygiene/clean deliveries, HIV/AIDS prevention, reduced deaths in the community and reduced criticisms of tTBAs by the community. Below the positive effects are explained in detail.

*“If a complication arises while at the clinic it’s easier for someone to contact the hospital unlike in our homes because some of us come from distant places and our roads are very bad”* (Respondent 4, FGD 1).

*“I think it has helped improve the hygiene in the community because when we were still delivering in the community we would just do it on the floor on a mat or sack with a plastic on top but at the clinic there are beds there and they clean those rooms better with chemicals. One time I delivered a woman from her kitchen outside because she was staying in a one room hut and the husband and other children were in the house at night. Now this is the same place where food is prepared so I think on that one it is a good idea to just go to the clinic.”* (30-year old tTBA).

“*On one side I would say there are no women and babies dying in the community. In the past we would hear of women dying in the community during labor but because of that decision we don’t hear of such deaths…”* (42-year old tTBA).

*“It helped us from criticisms from the community. For example, when a woman dies while in labor the community will just say that TBA never took good care of the woman besides why did she deliver someone who had complications she can’t handle. So government saved us very much”*(Respondent 2, FGD 2).

### Negative effects of the ban

The negative effects ranged from having extra work to do, loss of respect, recognition and feeling neglected, increased cost of lodging at the health facility, loss of social support from communities, to women getting back to untrained traditional birth attendants for child delivery.

*“…because of the Safe Motherhood Action Groups (SMAGS) I do a lot of walking escorting women to clinics far away from here but once there I do nothing at all”* (36-year old tTBA).

*“I don’t often go to the clinic though at first I would go frequently…pauses…..ah it’s like they don’t need us there anymore. Although the nurses don’t say it verbally but their actions show that am not needed. You can go there and they see you but they will just greet you and they get busy with whatever they are doing so I felt out of place most of the times and I stopped”* (30-year tTBA).

*“It’s rather annoying because they damped us. But what is surprising is that when they want something from us like information in the community they come back to us. So they just want to use us when they are stuck”* (Respondent 6, FGD 3).

*“I remember some time back people would organize themselves especially during the rainy season to come and cultivate the fields for me just as a way of appreciating what I used to do for their wives but since they heard that we were stopped by government they have stopped.”* (42-year old tTBA).

“*Staying at the relatives’ shelter entails running two separate homes. Women have to share the little food between those that remain at home and those that go to the clinic and this is expensive for us in the community. So we don’t know how government thought on that one because it never came to the people to explain.”* (Respondent 1, FGD 1).

*“…people that are not trained, the old women and relatives are the ones delivering in the community now.”* (45-year old tTBA).

## Discussion

The study showed that the ban has yielded both negative and positive results on the community. Some women have opted to continue delivering in the community despite the ban due to long distances to health facilities. This finding is similar to that of the Zambia Demographic and Health Survey of 2013/14 which showed that access to health facilities especially in rural areas was still a challenge [4]. Health facilities factors such as long distance to health facilities and limited skilled health workers and poor attitude of staff were some of the reasons for continued deliveries in the community. Regarding attitude, a study by Vogel also noted that globally many women experienced mistreatment during labor and child birth in health facilities which prevented some women from attending facility based deliveries [28].

In addition, social -cultural factors also negatively affected deliveries in health facilities. Some women did not accept clinic deliveries because they were culturally and traditionally used to delivering at home. This finding is in line with the study by Titaley who stated that health strategies involving TBAs were beneficial as they were traditionally and culturally appropriate [29].

Financial issues also negatively affected deliveries in health facilities. The study revealed that indirect costs associated with delivering at the health facility such as transport costs made some women delay in making the decision to go to the health facility services, a finding which is in conformity with the delays model in a study done in Zambia [23]. The health service coverage and evaluation model stresses that even when the health service is available, accessible and acceptable, its effective utilization depends equally on how affordable it is to the community [30].

Finally, limited community engagement in the decision making process and lack of effective communication of the decision to ban home deliveries also affected health facility deliveries. Traditional leaders and trained traditional birth attendants felt disrespected for failure by government to not only involve them in the decision making process but also not informing them officially about the decision and the reasons for it. The situation therefore highlights the role that community participation plays in shaping accessibility, acceptability and appropriateness of maternal and child health services at community level [20, 24].

Based on this study, we argue in line with Leedam [18], that the policy directives that only focus on one performance indicator (such as the policy on banning of TBAs in Zambia) without comprehensive consideration of wider health system, effects and possible options may not adequately support maternal health issues. One option for dealing with the situation of TBAs, as suggested by a study in Ethiopia, include providing comprehensive training of TBAs who are involved in the provision of pregnancy care in cases where TBAs are already frequently used by women [31]. We further recommend adoption of a stepwise approach to banning participation of TBAs. Adoption of a stepwise approach may provide possibilities for fully or regularly monitoring and documenting the positive and negative effects of the ban. Adopting of a stepwise approach is further important because it may provide opportunities for implementing strategies for increasing availability of midwives and health facilities before completely banning all the TBAs. Furthermore, a stepwise approach can help government develop measures for responding to some of the negative effects of the ban.

### Strengths and limitations of the study

The study employed three methods of data collection namely focus group discussions, in-depth interviews and key informant interviews. This use of multiple methods facilitated data triangulation.

Despite the above strength, this study had its own limitations inherent in the methodology. Firstly, generalizability of the findings of this study poses a critical problem. The study was conducted in one setting with a small sample of respondents taken from three (3) health facilities of Kazungula district yet the district has more than 22 health facilities. The findings may therefore not be representative of other settings. Similar studies are therefore warranted in other settings for comparability of research findings.

Secondly, the study only looked at the views of trained traditional birth attendants and some community leaders which do not give a full picture of the situation on the ground. There is need to triangulate the findings by looking at a larger source of information by getting the views of other stakeholders like health workers, men and women of childbearing age whose views are equally important.

## Conclusion

This study explored the experiences and effects of the ban of trained traditional birth attendants from conducting deliveries in a rural district. The study showed that home deliveries have continued despite the ban. Positive effects of the ban include early detection and management of pregnancy complications, enhanced HIV/AIDS prevention and better management of post-natal conditions, and reduced criticisms of tTBAs from the community in case of birth complications. Negatives effects of the ban include health facility related challenges such as extra work on the part of health workers, social-cultural factors such as loss of respect and recognition of TBAs by the community, some women not accepting clinic deliveries because they were culturally and traditionally used to delivering from their homes; as well as increased financial costs due to the need to travel and lodge at health facilities. Effectively managing the ban of tTBAs may require fully involving community stakeholders in policy formulation and dissemination. Furthermore, it is vital to consider adoption a step wise approach to implementing the ban of community deliveries as a rapid transition may trigger several negative consequences.
